# Effectiveness of obesity interventions in sub-Saharan Africa: A systematic review and meta-analyses

**DOI:** 10.1371/journal.pone.0323717

**Published:** 2025-05-23

**Authors:** Kingsley Agyemang, Shirley Crankson, Nana Anokye

**Affiliations:** 1 Department of Health Policy, School of Public Health, Management and Economics, Kwame Nkrumah University of Science and Technology (KNUST), Kumasi, Ghana; 2 Division of Global Public Health, Department of Health Sciences, College of Health, Medicine and Life Sciences, Brunel University London, Uxbridge, London, United Kingdom; Università degli Studi di Milano, ITALY

## Abstract

The escalating obesity epidemic in sub-Saharan Africa is a pressing regional concern. Despite this, there is scarce evidence of effective strategies to halt its upward trend in the region. We have, therefore, synthesised evidence on effective interventions to prevent and manage obesity in sub-Saharan Africa. We searched Scopus, PsycINFO, Cochrane Library, Web of Science and Medline for pertinent studies for this review. Studies were eligible if they focused on a sub-Saharan African country and assessed obesity/overweight with objective outcome measures. We examined their methodological quality with the Joanna Briggs Institute and the National Institutes of Health appraisal checklists. Publication bias was assessed with funnel plots. A meta-analysis with a random-effects model was fitted to explore the pooled effect of identified obesity interventions on anthropometric obesity measures. The heterogeneity of the studies was assessed using the I-square statistic. Our search yielded seven eligible studies for this review. Their quality ranged from moderate to high. The interventions identified included aerobic and resistance exercises, micronutrient supplementation and physical education. The meta-analysis revealed that aerobic and resistance training could significantly reduce obesity by approximately 34% (p = 0.04; 95%CI = -0.67 – -0.02). However, they do not significantly reduce waist circumference (Effect size = -1.14; 95%CI = -0.67–0.55; p = 0.19). Aerobic and resistance training exercises could be embedded in physical activity interventions to prevent and manage overweight and obesity in sub-Saharan Africa. PROSPERO registration number: CRD42023430503

## Introduction

Obesity is a significant health concern and a key risk factor for several chronic conditions, such as cardiovascular diseases, diabetes and cancers [1–3]. Since 1980, the obesity epidemic has increased exponentially, affecting about one-third of the world’s population [4]. More worrying is the growing burden of obesity in Low-Income Countries (LICs), including countries in sub-Saharan Africa (SSA), which used to experience a relatively lower prevalence of obesity [5–7], with the rates increasing in all ages and sexes [4,8], and more prevalent in urban populations [9,10]. For example, a study on the epidemiology of overweight and obesity in SSA showed that the prevalence of overweight in the region increased by more than 14%, from 6.4% in 1990 to 21% in 2015. In addition to the increasing trend, the obesity epidemic has also contributed to health disparities in SSA, with most health facilities and resource-deprived countries experiencing the brunt of the condition [6]. The rising levels of overweight and obesity in SSA are likely to exacerbate the burden of non-communicable diseases (NCDs) like Cardiovascular diseases (CVDs) and diabetes if effective strategies/interventions are not taken to mitigate the burden, given that they are risk factors for these NCDs [11].

Interventions to address obesity vary widely, with different approaches focusing on lifestyle changes, such as dietary changes and physical activity engagements [12]. However, the effectiveness of these interventions sometimes differs by population, given inherent characteristics such as socioeconomic diversities [13]. For example, a systematic review of obesity studies from Europe and the USA showed that combined diet and physical activity interventions do not influence the BMI of school-aged children [14], but a similar study with a similar study population in China found reverse results [15]. This variation in findings demonstrates how the outcomes of similar interventions could differ for similar populations from different socioeconomic contexts, suggesting the need for context-informed obesity interventions to address the global obesity menace.

Sub-Saharan countries have similar socioeconomic characteristics; therefore, synthesised evidence of obesity interventions from the region could have broader implications [16]. Current data on obesity interventions in SSA are mainly from primary studies [17–19], demonstrating literature scarcity on pooled evidence on effective obesity interventions [20–22] that could drive comprehensive obesity prevention and management strategies in SSA. Our study, therefore, addressed this gap by synthesizing the evidence on interventions to address obesity in SSA effectively. It specifically analysed obesity as the outcome, assessed with outcome measures like Body Mass Index (BMI), Waist Circumference (WC), and percentage (%) body fat.

## Methods

This review was guided by the PRISMA checklist (S1 Table) for reporting systematic reviews and meta-analyses. The review’s protocol is registered in PROSPERO (ID: CRD42023430503).

### Eligibility criteria

The following criteria were used to determine eligibility for this review:

Population: All human populations, including children, younger and older adults, men and women, were included in this review. Animal studies were excluded.

Interventions: We included all obesity interventions/programs/strategies aimed at reducing or preventing overweight or obesity in any sub-Saharan African country, comprising but not limited to single or combined dietary, physical activity and taxation policies, and environmental changes. It explored these interventions at all levels of engagement, including trial obesity/overweight interventions for large- or small-scale targeted populations and nationally or population/community-level obesity/overweight interventions. Accordingly, self-reported obesity interventions were excluded, given their tendencies to introduce biased findings as their authenticity may be unascertained/unconfirmed. Additionally, studies on clinical or surgical management of obesity were excluded as the review focused on public health interventions at a wider population level.

Comparator: No interventions or any measure of sedentary behaviour (e.g., no engagements in defined physical activities for a specified period) or dietary behaviours (e.g., any defined poor dietary habits, such as intake of highly caloric or sugar-sweetened foods for any measured period, like weekly, daily etc.

Outcomes: Outcomes included weight status, measured with objective obesity/overweight outcome measures, such as anthropometric and adiposity measures (e.g., BMI, WC and percentage body fat (% body fat)). Studies reporting weight outcomes based on self-reported anthropometric or adiposity were excluded.

Settings: We included all study settings located within a sub-Saharan country, such as but not limited to schools, churches, markets and corporate organisations.

Study type: The study types included any peer-reviewed quantitative, qualitative or mixed-methods studies published from the year 2000–2024 and were either experimental (including Randomized Controlled Trials (RCTs), quasi-experimental and pre-test post-test studies) or observational (including prospective and retrospective studies) study designs.

### Information sources

We comprehensively searched multiple databases, including Scopus, Medline, Web of Science, PsycINFO and the Cochrane Library for relevant studies for this review [23]. The search outcomes from the Cochrane Library also included reports from EMBASE, CINAHL and PubMed electronic databases. We also conducted reference tracking of the eligible papers from the electronic databases. The search was initially conducted from 1^st^ to 3^rd^ October 2023 and updated on 26^th^ July 2024.

### Search strategy

The search strategy encompassed Boolean operators combined with keywords derived from this review’s topic and aim and those from a similar study [24]. The combined keywords included ‘obesity’, ‘overweight’, ‘exercise’, ‘physical activity’, ‘diet’, ‘Africa’, ‘sub-Saharan Africa’, ‘tax’ and ‘effectiveness’. The search terms and outcomes for each included database are shown in S2 Table.

### Selection process

The titles and abstracts of the identified studies from the databases were downloaded in CSV/Microsoft Excel Spreadsheet and were screened independently by two reviewers (KA and SC) for inclusion in this review per the predefined eligibility criteria. Studies were agreed to be included in this review if they assessed or evaluated the effectiveness of a defined population-based obesity/overweight intervention or program in a sub-Saharan African country, countries that fall within the World Bank definition of sub-Saharan countries [25], shown in Table 1. Disagreements on the inclusion or exclusion of a paper were discussed and addressed with the other author (NA).

**Table 1:** Definition of sub-Saharan country.

Angola, Benin, Botswana, Burkina Faso, Burundi, Cabo Verde, Cameroon, Central African Republic, Chad, Comoros, Congo Dem. Rep., Congo Rep., Cote D’Ivoire, Equatorial Guinea, Eritrea, Eswatini, Ethiopia, Gabon, The Gambia, Ghana, Guinea, Guinea-Bissau, Kenya, Lesotho, Liberia, Madagascar, Malawi, Mali, Mauritania, Mauritius, Mozambique, Namibia, Niger, Nigeria, Rwanda, Sao Tome And Principe, Senegal, Seychelles, Sierra Leone, Somalia, South Africa, South Sudan, Sudan, Tanzania, Togo, Uganda, Zambia, Zimbabwe.

### Data collection process

We used a predetermined data extraction questions, informed by the JBI manual for evidence synthesis to extract data from the studies that met this review’s eligibility. The extractions were done independently and by two reviewers (KA and SC) for all the studies, and 50% of the extracted data were independently reviewed by a third reviewer (NA) to confirm the quality and extraction of all relevant data from the eligible studies.

### Data items

The extracted data items included the study’s authors, aims, title, year of publication, country/setting, design, study populations, sample characteristics (e.g., age and sex specifications and number), sample size, type and specifications of the intervention, duration of intervention, follow-up periods, measurement and specification of obesity, e.g., BMI, analytical estimators for assessing interventions’ effects, reported effect sizes and direction of effects and participant attrition. Authors or their relevant parent publications were contacted for any required data item that was missing in their studies.

### Risk of bias and quality assessment

We appraised the methodological quality of the included studies using the Joanna Briggs Institute (JBI) and the National Institutes of Health (NIH) appraisal checklists. The choice of these checklists was informed by a recent review examining the quality of quality appraisal tools [26]. We used specific JBI tools to appraise the quality of the RCTs and quasi-experimental studies, and the NIH tool for the quality assessment of the pre-test and post-test designs. On the JBI tool for the RCTs, studies that met ≥ 11 items on the tool were rated ‘high quality’, those meeting ≤5 were rated ‘low quality’, and those from 6 to 10 were rated moderate quality. For the quasi-experimental study, studies with yes responses to ≥7 items on the JBI tool were rated as ‘high quality’. Those ≤4 were ‘low quality’, and those from 5 to 6 as ‘moderate quality’. On the NIH, studies with yes scores ≥10 were ‘high quality’, those with ≤5 were ‘poor quality’, and those from 6 to 9 were ‘moderate quality’. The risk of bias of the RCTs was assessed with the Cochrane risk-of-bias tool for randomised trials (RoB 2), and that of the non-randomised studies was assessed with the Risk Of Bias In Non-randomised studies – of Interventions, Version 2 (ROBINS-I V2) tool. For both tools, studies that recorded low risk in all domains of the tools were rated ‘low risk of bias’, those with some concerns in some of the domains were rated ‘moderate risk of bias’, while those with significant methodological problems, as observed in many of the domains were rated ‘serious risk of bias’. We assessed publication bias with funnel plots [27,28]. Symmetrical funnels implied no publication bias, and the asymmetrical meant publication bias. KA and SC did the quality appraisal assessments, and any disagreements on the rated quality of the studies were discussed and addressed with NA.

### Data synthesis

A narrative data synthesis was first conducted to summarise the characteristics of the selected studies, including the studies’ population, sample size, effect sizes, follow-ups and obesity-related interventions and outcomes. Reported significances, effect sizes, and direction of influence were also synthesised narratively and compared for the studies with similar interventions and outcomes [29]. After the narrative synthesis, a meta-analysis was conducted using SPSS version 26 to examine the pooled effect of the obesity interventions on weight status. We also conducted a sub-group analysis to examine the influence of interventions per defined obesity outcome measures. A random-effects model was fitted to estimate the range of effect sizes, given the possible variation in the treatment effects of the studies due to potential differences in their intervention and outcomes circumstances [30,31]. Cohen’s d was used to estimate the standardised mean difference for each included study and between studies. Heterogeneity among the studies was assessed with the I^2^ and τ^2^ statistics. I^2^ results of >75% were interpreted as high heterogeneity, 50% to 75% as moderate heterogeneity, and those <50 as low heterogeneity. 95% Confidence Intervals (CI) were also used to supplement I^2^ and τ^2^ statistics by demonstrating the range of the effect estimate [32]. Forest plots were used to show the results of the pooled effects size and heterogeneity.

## Results

### Study selection

**Fig 1:**
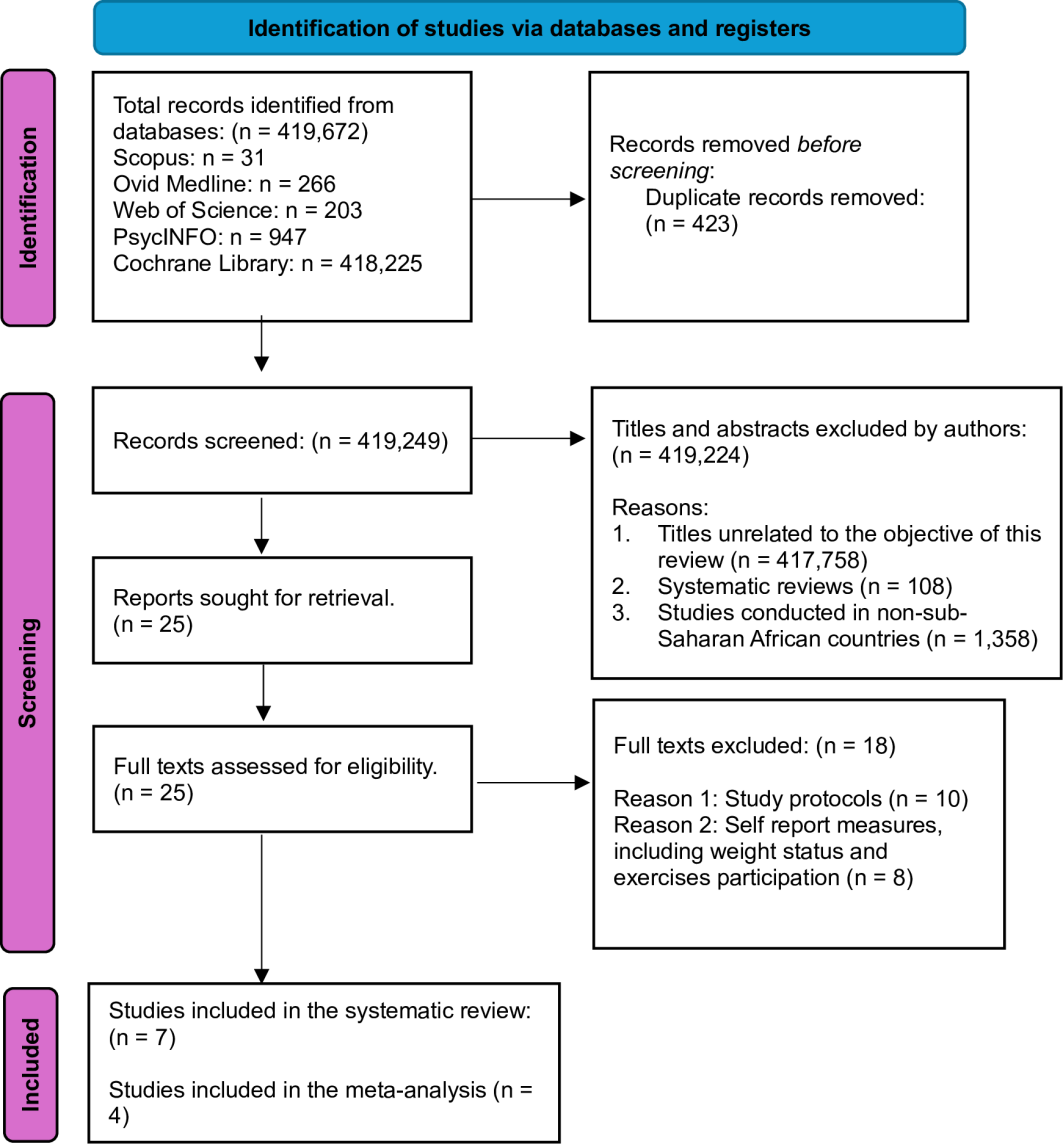
PRISMA flow diagram illustrating the studies selection process.

Of the 419,672 studies (S3 Table) identified through the database searches, 423 were duplicates, and 419,224 titles and abstracts were unrelated to this review. The full text of the remaining 25 were further screened, resulting in the removal of 18 additional studies that did not meet the eligibility criteria of this review. The selection process is shown in Fig 1.

### Study characteristics and findings

The selected seven studies were conducted in rural and urban settings in South Africa (SA) [17–19, 33–36]. They are all experimental research, with the majority as RCTs [18,19,34,36] and the rest as pre-test and post-test designs [17,35] and quasi-experimental [33]. The study population included school-aged children, college/university students and employees, corporate participants and church attendees. The total number of participants was 1,911, and more than half were female participants (n = 1,101; 57.6%). Some of the female participants (n = 160) were described as having obesity and another 40 had sedentary lifestyles. The participants were aged ≥6 years, with an estimated average age of 24.76 years (estimated from the average age data available/reported by 5 of the studies). On attrition, twelve out of the initially recruited 132 participants were missing in one of the studies [33], five in the control group and seven in the treatment group. These participants were lost at the start of the study and were excluded in the baseline and post-treatment analyses. Also, a little more than half of the eighty-four participants included in the baseline assessments of another study [17] were not included in the post-treatment WC (n = 42; 50%); BMI (n = 43; 51.2%) and HC (n = 45; 53.6%) outcome assessments.

The identified obesity interventions were exercises [17–19,33–36], education on weight loss benefits and micronutrient supplementation with tablets containing multivitamins [18]. The exercises were aerobics, with some studies describing it as walking and cycling exercises, and combined aerobic and resistance training exercises. The exercise interventions were delivered weekly, each lasting from 30 minutes to an hour. The duration of interventions ranged from six weeks to three years, with most of the studies having twelve weeks duration. None of the studies ended before the stipulated study duration. Interventions’ follow-up assessments were done at the end of the program, except for two studies that reported nine months and mid-program (duration not specified) follow-up assessments. Most of the studies (n = 5) used BMI and WC outcome measures [17,19,33–35], one used % body fat [36] and, another the bioelectrical impedance analysis for fat mass [18]. Other outcome measures used included WHR and Hip Circumference (HC) [17,33]. All the studies indicated that the intervention significantly reduced the weight status of the study population, except Gradidge and Golele [34], who found no significant association between the intervention and BMI and WC. None of the studies reported any undesired intervention outcomes and sustainability plans for the interventions for the defined populations. One of the included studies [19] had missing BMI and WC data, but the authors signposted to their parent publication [37], where the data were retrieved and included in this review. The characteristics and findings of the included studies are contained in S4 Table.

### Risk of bias and methodological quality of the studies

**Fig 2:**
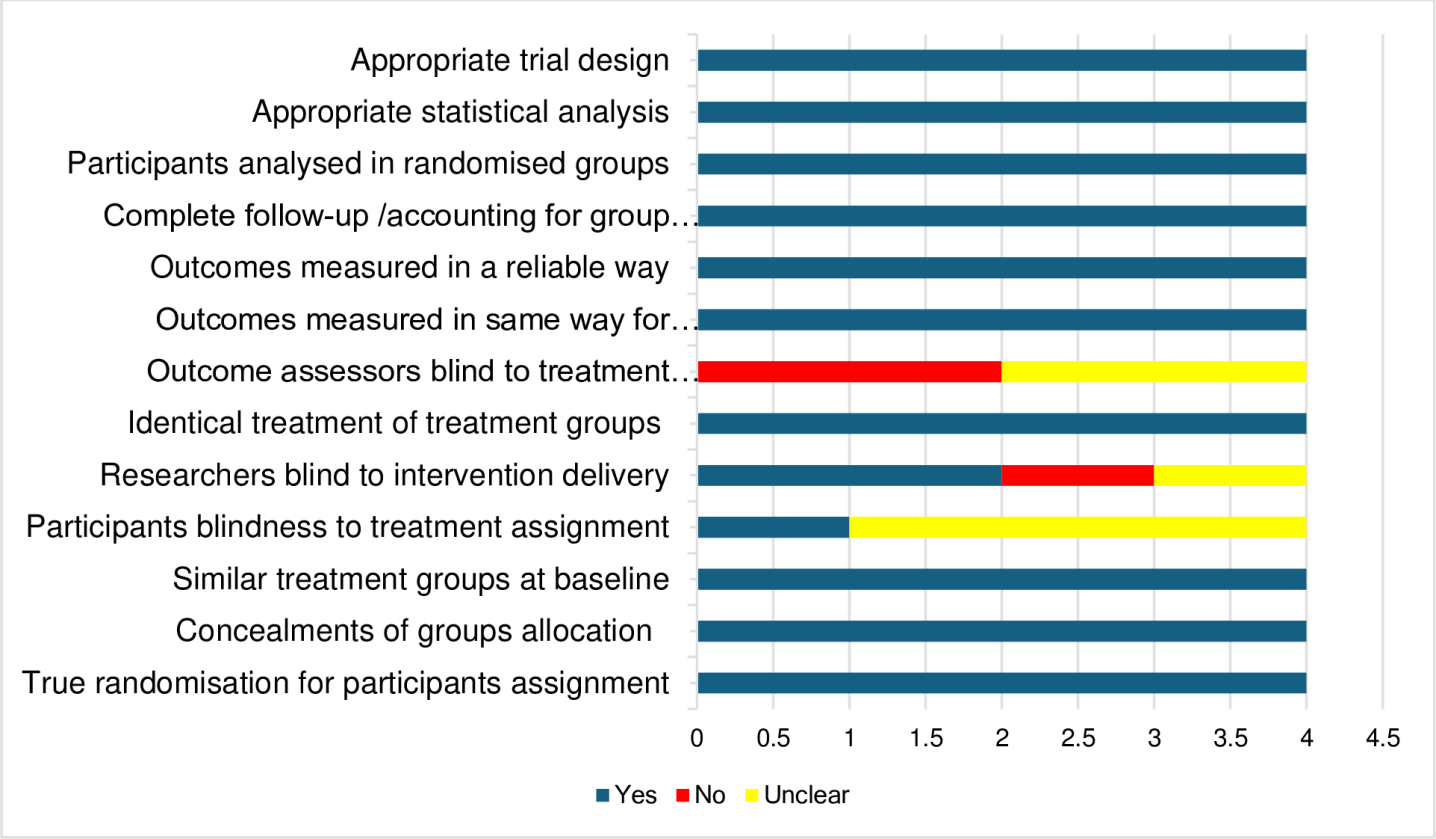
Summarised methodological quality of the RCTs with the JBI tool.

**Fig 3:**
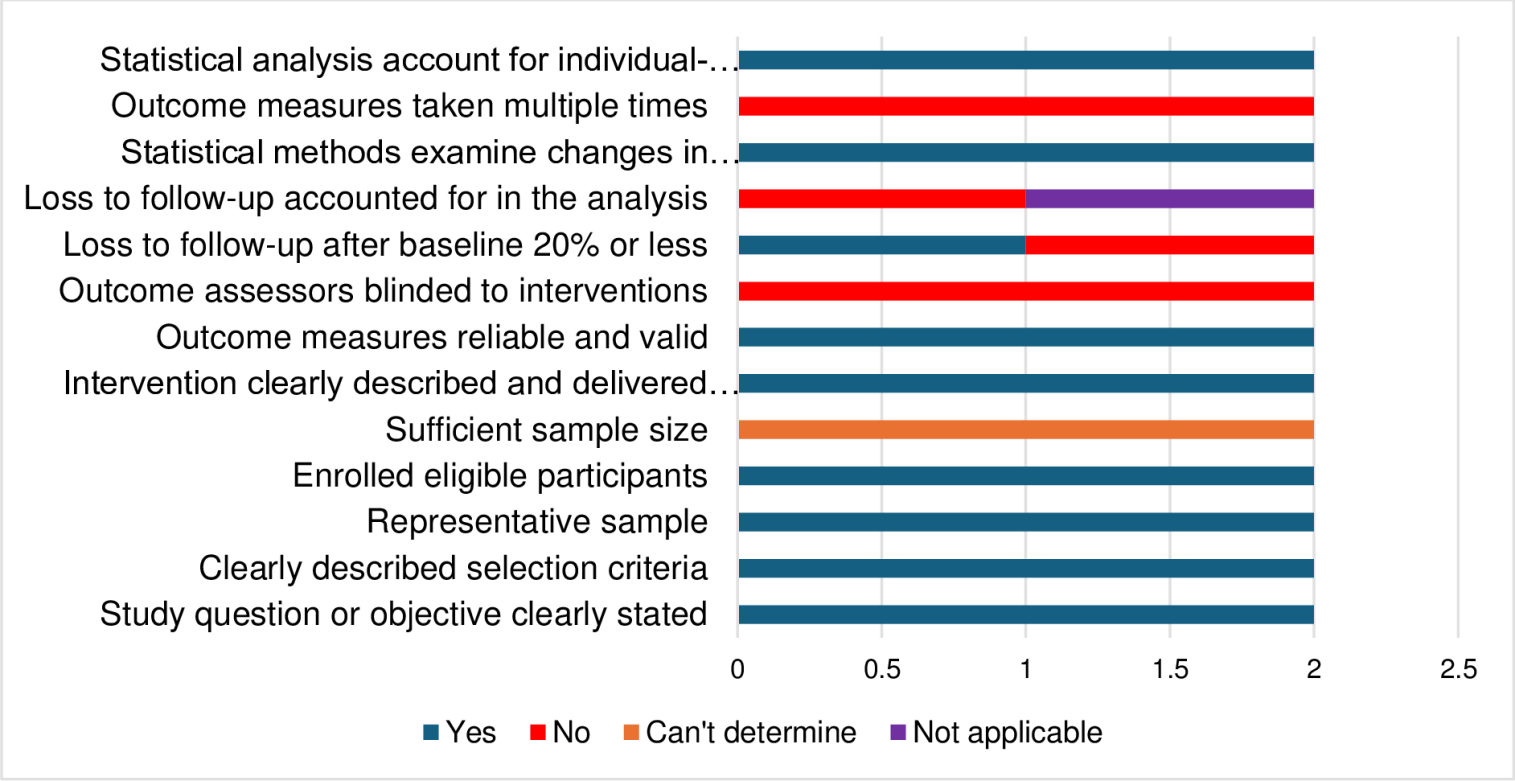
Summarised methodological quality of the pre-test-post-test studies using the NIH tool.

**Fig 4:**
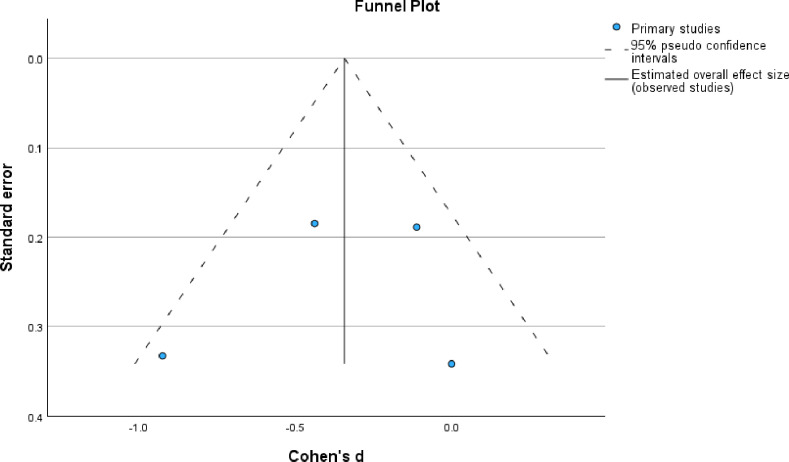
Symmetrical funnel plots showing no publication bias in the included studies.

The studies’ methodological qualities are detailed in Tables 2–4 and summarised in Figs 2 and 3, and the risk of bias results are shown in S5 Table. The publication bias is also shown in Fig 4. The methodological quality ranged from moderate to high across the used appraisal tools. The common methodological concerns in the RCTs were uncertainty on whether the participants were blind to their assigned groups (n = 3), uncertainty (n = 2) and no (n = 2) blindness of the outcome assessors to participants group assignments, and no (n = 1) and uncertainty (n = 1) on whether those delivering the treatment were blind to treatment assignment. For the pre-test-post-test designs, there were concerns associated with unreported statistical power (external validity), unblinded outcome assessors to participants’ intervention’s allocation, limited confidence in the accuracy of the measured outcomes, loss to follow-up (attrition) and not accounting for the loss-to-follow-up participants in the analysis. There was no identified methodological concern in the included quasi-experimental study. The studies recorded a low risk of bias on the RoB 2 and the ROBINS-I V2 tools, and there was also no publication bias as the funnel plot was symmetrical.

**Table 2:** Quality assessment results of the RCT studies using the JBI tool.

Criteria		Studies			
		Gradidge & Golele (2018)	Long et al (2022)	Nono Nankam et al. (2020)	Ntshaba et al. (2021)
**A. Bias related to selection and allocation**					
1	Was true randomization used for assignment of participants to treatment groups?	Yes	Yes	Yes	Yes
2	Was allocation to treatment groups concealed?	Yes	Yes	Yes	Yes
3	Were treatment groups similar at the baseline?	Yes	Yes	Yes	Yes
**B. Bias related to administration of intervention/exposure**					
4	Were participants blind to treatment assignment?	Unclear	Yes	Unclear	Unclear
5	Were those delivering the treatment blind to treatment assignment?	Unclear	Yes	Yes	No
6	Were treatment groups treated identically other than the intervention of interest?	Yes	Yes	Yes	Yes
**C. Bias related to assessment, detection and measurement of the outcome**					
7	Were outcome assessors blind to treatment assignment?				
	Outcome 1	No	Unclear	Unclear	No
	Outcome 2	No	–	Unclear	No
8	Were outcomes measured in the same way for treatment groups?				
	Outcome 1	Yes	Yes	Yes	Yes
	Outcome 2	Yes		Yes	Yes
9	Were outcomes measured in a reliable way				
	Outcome 1	Yes	Yes	Yes	Yes
	Outcome 2	Yes		Yes	Yes
**D. Bias related to participant retention**					
10	Was follow up complete and if not, were differences between groups in terms of their follow up adequately described and analysed?				
	Outcome 1	Yes	Yes	Yes	Yes
	Outcome 2	Yes		Yes	Yes
**E. Statistical conclusion validity**					
11	Were participants analysed in the groups to which they were randomized?				
	Outcome 1	Yes	Yes	Yes	Yes
	Outcome 2	Yes		Yes	Yes
12	Was appropriate statistical analysis used?				
	Outcome 1	Yes	Yes	Yes	Yes
	Outcome 2	Yes		Yes	Yes
13	F. Was the trial design appropriate and any deviations from the standard RCT design (individual randomization, parallel groups) accounted for in the conduct and analysis of the trial?	Yes	Yes	Yes	Yes
Quality rating Overall appraisal:		Moderate Include: ☒	High Include: ☒	High Include: ☒	Moderate Include: ☒

**Table 3:** Quality assessment results of the quasi-experimental study using the JBI tool.

Criteria		Study
		Mathunjwa et al. (2023)
1	Is it clear in the study what is the ‘cause’ and what is the ‘effect’ (i.e., there is no confusion about which variable comes first)?	Yes
2	Were the participants included in any comparisons similar?	Yes
3	Were the participants included in any comparisons receiving similar treatment/care, other than the exposure or intervention of interest?	Yes
4	Was there a control group?	Yes
5	Were there multiple measurements of the outcome both pre and post the intervention/exposure?	Yes
6	Was follow up complete?	Yes
	if not, were differences between groups in terms of their follow up adequately described and analysed?	Not applicable
7	Were the outcomes of participants included in any comparisons measured in the same way?	Yes
8	Were outcomes measured in a reliable way?	Yes
9	Was appropriate statistical analysis used?	Yes
Quality rating Overall appraisal:		High Include: ☒

**Table 4:** Quality assessment results of the pre-test post-test studies using the NIH tool.

Criteria		Studies	
		Draper et al. (2019)	Torres et al. (2020)
1	Was the study question or objective clearly stated?	Yes	Yes
2	Were eligibility/selection criteria for the study population prespecified and clearly described?	Yes	Yes
3	Were the participants in the study representative of those who would be eligible for the test/service/intervention in the general or clinical population of interest?	Yes	Yes
4	Were all eligible participants that met the prespecified entry criteria enrolled?	Yes	Yes
5	Was the sample size sufficiently large to provide confidence in the findings?	Cannot determine	Cannot determine
6	Was the test/service/intervention clearly described and delivered consistently across the study population?	Yes	Yes
7	Were the outcome measures prespecified, clearly defined, valid, reliable, and assessed consistently across all study participants?	Yes	Yes
8	Were the people assessing the outcomes blinded to the participants’ exposures/interventions?	No	No
9	Was the loss to follow-up after baseline 20% or less?	No	Yes
	Were those lost to follow-up accounted for in the analysis?	No	Not applicable
10	Did the statistical methods examine changes in outcome measures from before to after the intervention?	Yes	Yes
11	Were outcome measures of interest taken multiple times before the intervention and multiple times after the intervention (i.e., did they use an interrupted time-series design)?	No	No
12	If the intervention was conducted at a group level (e.g., a whole hospital, a community, etc.) did the statistical analysis take into account the use of individual-level data to determine effects at the group level?	Yes	Yes
Overall rating		Moderate	Moderate

### Effect of exercise interventions on anthropometric measures

**Fig 5:**
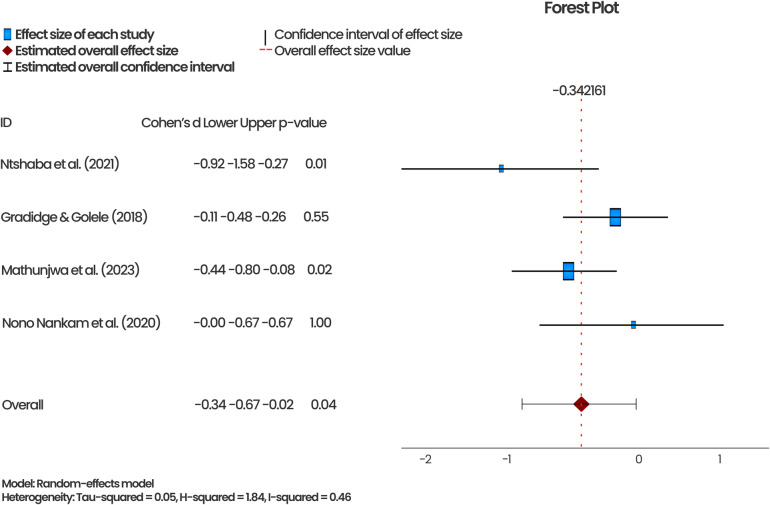
Effect of aerobic and resistance training exercise on weight status.

**Fig 6:**
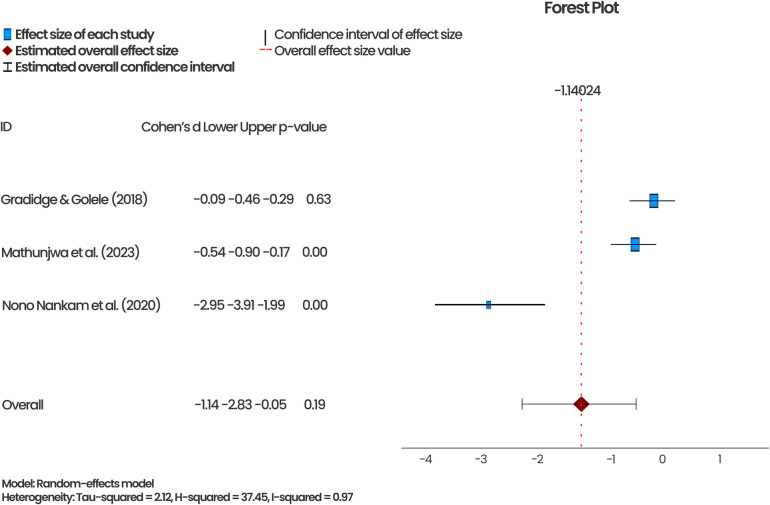
Effect of aerobic and resistance training on Waist Circumference.

Figs 5 and 6 show the pooled effect of exercise interventions (aerobic and resistance training) on weight status and WC, respectively, from four of the RCTs that were eligible for the meta-analysis. The meta-analysis showed that the exercise interventions significantly reduced overweight among the study populations by 34% (p = 0.04; 95%CI = -0.67 – -0.02). An I^2^ of 46% from the pooled analysis indicated a low heterogeneity among the studies included in this meta-analysis. The effect sizes of the three similar studies assessing exercise intervention and WC ranged from -0.09 to -2.95. Their combined effect size was -1.14, with a p-value of 0.19, indicating that the intervention reduced WC; however, the reduction was insignificant. In addition, the 95%CI contained ‘0’ (-0.67–0.55), suggesting that the identified effect may not be attributable to the exercise intervention. The I^2^ of 97% indicate high variations in the included studies.

## Discussion

Our study aimed to address the paucity of literature on effective interventions to address obesity in SSA. We identified and included seven relevant studies after a comprehensive literature search using multiple databases such as Cochrane Library, Web of Science and PsycINFO. All the relevant studies were from South Africa, highlighting a literature scarcity on effectiveness of obesity interventions studies from other SSA countries and demonstrating the need for such studies to ensure representative evidence on the research area in SSA. Notwithstanding, it is possible that obesity intervention studies from other SSA countries are contained in databases that are different from those included in this review.

We found out from the meta-analysis that aerobic and resistance training exercises could significantly reduce overweight/obesity by more than 30%. This finding was consistent with similar meta-analyses in the literature, which showed that aerobic and resistance exercises could reduce BMI, fat mass and %body fat among children and adolescents with overweight/obesity [38,39]. Other meta-analyses demonstrate that aerobic exercises are central for exercise interventions aimed at reducing adiposity among the adult populations [40]; however, combining it with resistance training could significantly optimise the impact of the exercise intervention [41]. Accordingly, future exercise intervention prescriptions to address obesity in SSA could explore combined aerobic and resistance training, as this review and evidence from the literature support their effectiveness. We could not compare our findings to similar meta-analyses from SSA to generalise and draw a more reliable conclusion about the efficacy of combined aerobic and resistance training exercises to address obesity in SSA because of the scarcity of such evidence synthesis in the defined study area. However, our finding was confirmed in a comparable narrative review, which reported that physical activity interventions could prevent childhood obesity in Africa [42].

We did not find any significant influence of the aerobic and resistance training on WC anthropometric measure. We could not also attribute the identified reduction in WC in the meta-analysis to the effect of the exercise intervention, given the inclusion of zero in the confidence interval. This attribution limitation could perhaps be stemming from the high heterogeneity among the studies included in that sub-group analysis, which varied in terms of participant demographics, intervention duration, and outcome measures, limiting this study from concluding on the effectiveness of aerobic and resistance training on WC [43]. However, given the documented evidence from similar meta-analysis in the literature, aerobic and resistance exercise could be explored by clinicians and policymakers as mitigating interventions to address overweight and obesity in SSA [44,45]. We could not also conclude on the effectiveness of the other identified obesity mitigating interventions: micronutrient supplementation and physical education because of their limited data in this review. More primary studies on these interventions in SSA may be necessary to provide synthesised evidence on their effectiveness in preventing and managing overweight/obesity in SSA.

Our study addressed the scarcity of research on effective interventions to manage obesity in SSA. Our findings, therefore, provide additional knowledge to direct intervention decisions to curb the obesity epidemic in the region. We did not identify publication bias, and the methodological quality of the included studies was moderate to high, potentially influencing the quality of this review. However, we are limited in generalising the implications of our findings to all countries in SSA, given that the included studies were all from South Africa. This limitation was, however, due to the observed unavailability of such studies from the other SSA countries in the current literature, emphasising the urgent research need for these studies in these countries. There were also high variations in the studies included in the sub-group analyses on WC, limiting conclusions on the effect of the interventions on WC. Further, the review focused on only public health interventions, excluding surgical and clinical interventions. This exclusion limits the scope of the review and the inferences of the findings for clinical/surgical obesity management. In using SPSS for the meta-analysis, we acknowledge that it may employ slightly different default estimation techniques from other software like STATA, which could lead to minor variations in results. However, we ensured that our methods aligned with established meta-analysis practices to allow replication of our findings.

## Conclusions

Aerobic and resistance training exercises are correlated with reductions in overweight and obesity anthropometric measures. Combining these interventions in an exercise program could maximise their benefits in preventing and managing obesity. Therefore, health practitioners in SSA could explore the combination of these interventions in obesity management strategies. It is recommended that countries in SSA conduct country-specific research on effective obesity interventions to contribute to collaborative efforts at managing the obesity epidemic in the region.

## Supporting information

S1 Table PRISMA checklist. (PDF)

S2 Table Databases search outcomes. (PDF)

S3 Table List of all identified studies from the literature search. (XLSX)

S4 Table Data extraction table. (XLSX)

S5 Table Risk of bias assessment results. (PDF)
