# Supplementary material for: Effectiveness of obesity interventions in sub-Saharan Africa: A systematic review and meta-analyses
Source: PLoS One. 2025 May 23;20(5):e0323717. doi: 10.1371/journal.pone.0323717 (PMC12101849; doi:10.1371/journal.pone.0323717)
Supplement: S2 Table — (PDF) [file pone.0323717.s002.pdf]

Databases search outcomes

Databases	Search strategy	Initial hit	Filters applied (numbers remaining after applying filter)	Final hit
Scopus	obesity OR overweight AND exercise OR activity OR food OR diet OR subsidy OR taxation OR tax OR intervention AND effectiveness AND Africa	80	• Papers from 2000 – 2024 (n = 78)<br>• Open access papers (n = 62)<br>• Limit to research articles (n = 38)<br>• Limit to studies on populations/countries from sub-Saharan Africa (n = 31)	31
Medline (Ovid)	Obesity or overweight AND exercise or activity OR food OR diet OR subsidy OR taxation OR tax OR intervention AND effectiveness AND Africa	1,641	• Papers from 2000 – 2024 (n = 1,494)<br>• Limit to full-text papers (n = 276)<br>• Limit to human's studies (n = 266)	266
Web of Science	obesity OR overweight AND exercise OR activity OR food OR diet OR subsidy OR taxation OR tax OR intervention AND effectiveness AND Africa	8,446,545	• Used the 'refine search results box' to search results of the initial hit with the words: 'obesity', overweight', 'intervention', 'effectiveness' 'efficacy' (n = 343).<br>• Papers from 2000 – 2024 (n = 343)<br>• Limit to open access papers (n = 259)<br>• Limit to research articles (n = 205)<br>• Limit to articles published in the English Language (n = 203)	203
PsycINFO	obesity OR overweight OR obese OR unhealthy weight OR high BMI AND interventions OR programs OR strategies AND	17,114	• Limit to Papers from 2000 – 2024 (16,252)<br>• Limit to open access (n = 1900)<br>• Limit to full-text available (n = 969)	947

Databases	Search strategy	Initial hit	Filters applied (numbers remaining after applying filter)	Final hit
	effectiveness OR efficacy OR effective OR success OR outcome OR evaluation OR analysis OR impact		• Limit to articles published in the English Language (n = 947)	
Cochrane Library (Title and abstract keyword) (advanced search	obesity OR overweight AND exercise OR activity OR food OR diet OR subsidy OR taxation OR tax OR intervention AND effectiveness AND Africa	484,914 (Trials)	• Limit to articles from 2000 – 2024<br>• (n = 428,025)<br>• Limit to articles published in the English Language (n = 418,225)	418,225
Total				419,672
