# Supplementary material for: Effectiveness of obesity interventions in sub-Saharan Africa: A systematic review and meta-analyses
Source: PLoS One. 2025 May 23;20(5):e0323717. doi: 10.1371/journal.pone.0323717 (PMC12101849; doi:10.1371/journal.pone.0323717)
Supplement: S5 Table — (PDF) [file pone.0323717.s005.pdf]

Risk of bias assessment results of the RCTs using Cochrane risk-of-bias tool for randomised trials (RoB 2)

Domains		Studies			
		Gradidge & Golele (2018)	Long et al (2022)	Nono Nankam et al. (2020)	Ntshaba et al. (2021)
**1. Bias arising from the randomisation process**					
1.1	Was the allocation sequence random?	Possibly Yes (PY)	PY	PY	PY
1.2	Was the allocation sequence concealed until participants were enrolled and assigned into interventions?	PY	PY	PY	PY
1.3	Did baseline differences between groups suggest a problem with the randomisation process	No	No	No	No
Risk of bias judgement		Low risk (LR)	LR	LR	LR
**2. Bias due to deviations from intended interventions (effect of assignment to intervention)**					
2.1	Were participants aware of their assigned intervention during the trial?	No information (NI)	No	NI	NI

Domains		Studies			
		Gradidge & Golele (2018)	Long et al (2022)	Nono Nankam et al. (2020)	Ntshaba et al. (2021)
2.2	Were carers and people delivering the interventions aware of participants' assigned intervention during the trial?	NI	No	No	Yes
2.3	If Yes/Probably Yes/NI to 2.1 or 2.2, Were there deviations from the intended intervention that arose because of the trial context?	No	Not applicable (NA)	NA	No
2.4	If Y/PY to 2.3: Were these deviations likely to have affected the outcome?	NA	NA	NA	NA
2.5	If Y/PY/NI to 2.4: Were these deviations from intended intervention balanced between groups?	NA	NA	NA	NA
2.6	Was an appropriate analysis used to estimate the effect of assignment to intervention?	Yes	Yes	Yes	Yes
Risk of bias judgement		LR	LR	LR	LR

Domains		Studies			
		Gradidge & Golele (2018)	Long et al (2022)	Nono Nankam et al. (2020)	Ntshaba et al. (2021)
**Bias due to deviations from the intended interventions (effect of adhering to intervention)**					
2.1	Were participants aware of their assigned intervention during the trial?	NI	No	NI	NI
2.2	Were carers and people delivering the interventions aware of participants' assigned intervention during the trial?	NI	No	No	Yes
2.3	[If applicable:] If Y/PY/NI to 2.1 or 2.2: Were important non-protocol interventions balanced across intervention groups?	NA	NA	NA	NA
2.4	[If applicable:] Were there failures in implementing the intervention that could have affected the outcome?	NA	NA	NA	NA
2.5	[If applicable:] Was there non-adherence to the assigned	NA	NA	NA	NA

Domains		Studies			
		Gradidge & Golele (2018)	Long et al (2022)	Nono Nankam et al. (2020)	Ntshaba et al. (2021)
	intervention regimen that could have affected participants' outcomes?				
Risk of bias judgement		LR	LR	LR	LR
**3. Bias due to missing outcome data**					
3.1	Were data for this outcome available for all, or nearly all, participants randomised?	Yes	Yes	Yes	Yes
Risk of bias judgement		LR	LR	LR	LR
**4. Bias in measurement of the outcome**					
4.1	Was the method of measuring the outcome inappropriate?	No	No	No	No
4.2	Could measurement or ascertainment of the outcome have differed between intervention groups?	No	No	No	No
4.3	If No/Possibly No (PN)/NI to 4.1and 4.2: Were outcome assessors aware of the	Yes	PY	PY	Yes

Domains		Studies			
		Gradidge & Golele (2018)	Long et al (2022)	Nono Nankam et al. (2020)	Ntshaba et al. (2021)
	intervention received by study participants?				
4.4	If Yes/PY/NI to 4.3: Could assessment of the outcome have been influenced by knowledge of intervention received?	PN	PN	PN	PN
Risk of bias judgement		LR	LR	LR	LR
**5. Bias in selection of the reported result**					
5.1	Were the data that produced this result analysed in accordance with a pre-specified analysis plan that was finalized before unblinded outcome data were available for analysis?	PY	PY	PY	PY
5.2	Is the numerical result being assessed likely to have been selected, on the basis of the results, from multiple eligible outcome measurements (e.g.	No	No	No	No

Domains		Studies			
		Gradidge & Golele (2018)	Long et al (2022)	Nono Nankam et al. (2020)	Ntshaba et al. (2021)
	scales, definitions, time points) within the outcome domain?				
5.3	Is the numerical result being assessed likely to have been selected, on the basis of the results, from multiple eligible analyses of the data?	No	No	No	No

Risk of bias assessment results of the non-randomised studies using the Risk Of Bias In Non-randomised studies – of Interventions, Version 2 (ROBINS-I V2) assessment tool

Criteria		Studies		
		Draper et al. (2019)	Torres et al. (2020)	Mathunjwa et al. (2023)
**1. A. Bias due to confounding (Domain 1, Variant A (only baseline confounding needs to be addressed – if N to C2, or Y to C2 and N to C3))**				
1.1	Did the authors control for all the important confounding factors for which this was necessary?	Yes	Yes	Yes
1.2	If Y/PY/WN to 1.1: Were confounding factors that were controlled for (and for which control was necessary) measured validly and reliably by the variables available in this study?	Yes	Yes	Yes
1.3	If Y/PY/WN to 1.1: Did the authors control for any post-intervention variables that could have been affected by the intervention?	NI	NI	NI
1.4	Did the use of negative controls, quantitative bias analysis, or other considerations, suggest serious unmeasured confounding?	No	No	No
Risk of bias judgement		Low risk (LR)	LR	LR
**2. Bias in classification of interventions**				

Criteria		Studies		
		Draper et al. (2019)	Torres et al. (2020)	Mathunjwa et al. (2023)
2.1	Did assignment of participants to the intervention group or the comparator group rely on events or measurements that occurred after the start of follow up?	NA	NA	No
2.2	If Y/PY to 2.1: Were participants included in the comparator group until they fulfilled the definition of the intervention (or vice versa)?	NA	NA	NA
2.3	If N/PN to 2.1: Was all information used to classify intervention and comparator groups recorded at or before the time the interventions started?	NA	NA	NA
2.4	Was classification of intervention status influenced by knowledge of the outcome or risk of the outcome?	NA	NA	No
2.5	If N/PN to 2.1 and WY/N/PN/NI 2.4: Was intervention status classified correctly for all, or nearly all, participants?	NA	NA	NA
Risk of bias judgement		LR	LR	LR
**3. Bias in selection of participants into the study (or into the analysis)**				

Criteria		Studies		
		Draper et al. (2019)	Torres et al. (2020)	Mathunjwa et al. (2023)
3.1	Did assignment of participants to the intervention group or the comparator group rely on events or measurements that occurred after the start of follow up?	NA	NA	No
3.2	If Y/PY to 3.1: Were participants excluded after the start of follow-up because they did not meet the definition of either the intervention or the comparator?	NA	NA	NA
3.3	Were start of follow up and start of intervention the same for most participants?	Yes	Yes	Yes
3.4	If N/PN to 3.3: Is the effect of intervention expected to be constant over the time period studied?	NA	NA	NA
3.5	Was selection of participants into the study (or into the analysis) based on participant characteristics observed after the start of intervention (additional to the situations addressed in 3.1 and 3.3)?	No	No	No
Risk of bias judgement		LR	LR	LR
**4. Bias due to deviations from intended interventions (Effect of assignment to intervention)**				

Criteria		Studies		
		Draper et al. (2019)	Torres et al. (2020)	Mathunjwa et al. (2023)
4.1	Was the study undertaken in an experimental context?	Yes	Yes	Yes
4.2	If Y/PY to 4.1: Did participants deviate from the intended intervention as a result of the processes of recruiting and engaging them in the study?	No	No	No
4.3	If Y/PY to 4.1: Did study personnel consciously or unconsciously undermine implementation of the intended interventions?	No	No	No
4.4	If Y/PY/NI to 4.2 or 4.3: Were these deviations from intended intervention likely to have affected the outcome?	NA	NA	NA
4.5	Was an appropriate analysis used to estimate the effect of assignment to intervention?	Yes	Yes	Yes
Risk of bias judgement		LR	LR	LR
**4. Bias due to deviations from intended interventions (effect of adhering to intervention)**				
4.1	Did all or nearly all participants adhere to their assigned intervention strategy?	Yes	Yes	Yes

Criteria		Studies		
		Draper et al. (2019)	Torres et al. (2020)	Mathunjwa et al. (2023)
4.2	If N/PN/NI to 4.1: Were the protocol deviations likely to have affected the outcome?	NA	NA	NA
4.3	If Y/PY to 4.2: Was an appropriate analysis used to estimate the specified per-protocol effect, accounting for the specified protocol deviations?	NA	NA	NA
Risk of bias judgement		LR	LR	LR
**5. Bias due to missing data**				
5.1	Were complete data on intervention status available for all, or nearly all, participants?	Yes	Yes	Yes
5.2	Were complete data on the outcome available for all, or nearly all, participants?	Yes	Yes	Yes
5.3	Were complete data on important confounding variables available for all, or nearly all, participants?	Yes	Yes	Yes
5.4	if N/PN/NI to 5.1, 5.2 or 5.3: Is the result based on a complete case analysis?	NA	NA	NA
5.5	If Y/PY/NI to 5.4: Was exclusion from the analysis because of missing data (in intervention, confounders or the outcome)	NA	NA	NA

Criteria		Studies		
		Draper et al. (2019)	Torres et al. (2020)	Mathunjwa et al. (2023)
	likely to be related to the true value of the outcome?			
Risk of bias judgement		LR	LR	LR
**6. Bias in measurement of the outcome**				
6.1	Could measurement or ascertainment of the outcome have differed between intervention groups?	No	No	No
6.2	Were outcome assessors aware of the intervention received by study participants?	Yes	Yes	Yes
6.3	If Y/PY/NI to 6.2: Could assessment of the outcome have been influenced by knowledge of the intervention received?	PN	PN	PN
Risk of bias judgement		LR	LR	LR
**7. Bias in selection of the reported result**				
7.1	Was the result reported in accordance with an available, pre-determined analysis plan?	Yes	Yes	Yes
7.2	Is the numerical result being assessed likely to have been selected, on the basis of the results, from multiple outcome	No	No	No

Criteria		Studies		
		Draper et al. (2019)	Torres et al. (2020)	Mathunjwa et al. (2023)
	measurements (e.g. scales, definitions, time points) within the outcome domain?			
7.3	Is the numerical result being assessed likely to have been selected, on the basis of the results, from multiple *analyses* of the data?	No	No	No
7.4	Is the numerical result being assessed likely to have been selected, on the basis of the results, from multiple *subgroups*?	No	No	No
Risk of bias judgement		LR	LR	LR
Overall risk of bias		LR	LR	LR

PY – Possibly Yes; PN – Possibly No; WN (no, but uncontrolled confounding was probably not substantial); SN (no, and uncontrolled confounding was probably substantial); NI – No Information; NA – Not Applicable; WY (yes, but the impact was not substantial)
